# Health-Related Social Needs and Total Healthcare Cost: A Cross-Sectional Study in a Large Integrated Health System

**DOI:** 10.1007/s11606-025-09647-8

**Published:** 2025-06-25

**Authors:** Ariel R. C. Silverman, Paul J. Chung, Michael K. Gould, Quyen Ngo-Metzger, Maile M. Tauali’i, David M. Mosen, Mark C. Duggan, Robert S. Nocon

**Affiliations:** 1 Kaiser Permanente Bernard J. Tyson School of Medicine, Pasadena, CA USA; 2 Department of Research and Evaluation, Kaiser Permanente Southern California, Pasadena, CA USA; 3 Hawaii Permanente Medical Group, Honolulu, HI USA; 4 Kaiser Permanente Center for Health Research, Portland, OR USA; 5 Present Address: Department of Internal Medicine, University of California, Los Angeles, Los Angeles, CA USA

**Keywords:** Health-related social needs, Social determinants of health, Social drivers of health, Social risk, Healthcare cost

## Abstract

**Background:**

While research on health-related social needs (HRSNs) has expanded, important gaps remain in understanding associations between HRSN and healthcare cost, especially across general populations of patients with broad-ranging medical and social needs.

**Objective:**

To examine the association between HRSN and healthcare cost in a large, diverse, insured population.

**Design:**

In this cross-sectional study, we evaluated cost differences between patients with different HRSN levels using survey-weighted multivariable generalized linear models. We examined three alternate model specifications: one that included controls for basic demographics; another adding insurance type, race/ethnicity, and social isolation; and a third adding a diagnosis-based medical risk index called DxCG. Variables added in the latter models were assessed separately due to concern for over-correlations with HRSN.

**Participants:**

10,226 adult survey respondents (23% response rate) from eight states and Washington, D.C.

**Main Measures:**

The primary exposure was patient-reported HRSN, based on survey questions asking about financial strain, food insecurity, housing instability, and transportation difficulties. HRSN was constructed as a three-level variable. The primary outcome was total direct healthcare cost.

**Key Results:**

“Moderate HRSN” was not significantly associated with cost in any model. In the model controlling for patient demographics, costs for patients with “Severe HRSN” were 1.27 (95%CI: 1.00–1.60) times those of patients with “No HRSN.” In a model that adjusted for medical risk (DxCG), the relationship between HRSN and cost was not statistically significant.

**Conclusions:**

Relationships between HRSN and healthcare cost may vary by HRSN level. Our mixed findings highlight the complex relationship between medical and social risks, which often have bi-directional causal relationships. If measures of medical risk incidentally capture variation in social risk, then models controlling for medical risk may mask relationships between HRSN and cost. Further studies should investigate the extent to which HRSN may be related to cost, even when controlling for specific patient diagnoses.

**Supplementary Information:**

The online version contains supplementary material available at 10.1007/s11606-025-09647-8.

## INTRODUCTION

Social determinants of health (SDoH) are the community-level conditions in which people are born, grow, work, live, and age, and they consist of a broad set of non-medical factors that may influence health outcomes^1^. Health-related social needs (HRSNs) operate at the individual level and refer to the social and economic needs that individuals experience resulting from underlying social determinants. HRSNs often contribute to adverse health outcomes and disparities^2–4^. As the importance and impact of HRSNs are increasingly recognized, efforts have grown by healthcare systems, payers, and other organizations to identify patients who may benefit from social health interventions. However, gaps remain in understanding the distribution of social needs in the general population, ideal patient populations for whom interventions should be targeted, and potential returns on investment of such interventions.

HRSN are widely believed to contribute to healthcare costs through their adverse impact on access to care, lifestyle-related risk factors, and overall health status, among other factors. However, the association between HRSN and cost has not yet been firmly established in a general population of patients. Existing research has largely focused on specific needs (e.g., housing)^5^, narrow populations (e.g., veterans)^6^, small-scale interventions^7,8^, specific disease outcomes^9–11^, populations with high levels of disease burden^12,13^, or some combination thereof. Additionally, much of the research has focused on Medicare beneficiaries, and research in more diverse populations is sparse^15–18^. Research is also challenged by a lack of standardization in screening and documentation for HRSN, and much of the existing work uses provider-entered Z-codes (a subcategory of the ICD-CM system for coding HRSN/SDoH), which are historically under-reported and poorly reflective of need burden^14,[19–22]^. Thus, there is a paucity of studies evaluating broad-based social needs screening and associated outcomes.

This study explored associations between an individual’s HRSN and their healthcare costs in an organization-wide sample of Kaiser Permanente (KP) members using data from a national survey combined with administrative data. KP is one of the nation’s largest integrated health systems, serving 12.5 million members. KP members included in this study comprise a broad range of insurance types, including Medicare, Medicaid, employer-sponsored insurance, and those who purchase individual insurance policies. As an integrated system, KP serves as both a payer and provider of a broad range of health services across the care spectrum. The study adds to the existing literature by exploring relationships between a broad set of patient-reported HRSN and total healthcare cost in an insured sample of patients that is diverse with respect to insurance type, age, sex, race/ethnicity, education level, and household income^23^. By studying outcomes in a large, integrated healthcare system, estimates can be made on a population level, which has useful implications for approaching larger health policy.

## METHODS

### Survey Methodology

This cross-sectional study utilized data from the 2020 Social Needs Network for Evaluation and Translation (SONNET) National Social Needs Survey, which is part of a broader effort to develop and evaluate HRSN-focused quality improvement initiatives^24,25^. For example, the survey was recently used in a study evaluating the association between HRSN and colorectal cancer screening rates^26^. The survey was distributed to 43,936 adult members from all eight KP regions: Northern California, Southern California, Colorado, Georgia, Hawaii, Mid-Atlantic (Maryland, Washington D.C., and Virginia), Washington, and Northwest (Oregon and southern Washington). The survey aimed to understand the prevalence of social needs known to affect health and wellbeing^22^^,23^. It was administered in January and September of 2020 in 26 waves. Participants were first contacted by email (if available) and letter, followed by email reminders, paper surveys, and follow-up calls if they did not respond.

An equal number of individuals were sampled in each KP market to ensure adequate sample size for within-region analysis of results. Stratified sampling was also done within each region to match regional age-sex distribution. Emphasis was placed on potentially vulnerable members by including a higher proportion of members residing in census blocks with median household incomes at or below the 25th percentile for that region, recent Medicaid enrollees, and recent applicants for medical financial assistance. Survey responses were weighted to be representative of the national KP population and to account for oversampling and non-response. The KP Washington Research Institute’s Institutional Review Board (IRB) designated the study as exempt from full review, given that it was part of a quality improvement effort and thus determined to be “Not Human Subjects Research.”

### Exposure

The primary exposure was overall HRSN, and secondary exposures included four domains of HRSN: financial instability, food insecurity, housing instability, and transportation needs. The 2020 survey asked respondents to reflect on HRSN over the prior year. For each HRSN domain, the level of need was categorized as “No Need,” “Moderate Need,” or “Severe Need” based on the level of response to Likert-style questions. Appendix 1 shows the questions and response options corresponding to the categorization of an individual as having “Moderate” or “Severe” need. An individual was categorized as having “Severe Need” for a specific domain if they indicated a response that corresponded to a severe need for any question within that domain. If they indicated a need at a level not qualifying as severe, they were categorized as having “Moderate Need” in that domain. Based on the need level for each of the four domains, an overall HRSN level was assigned such that those with a severe need in *any* domain were categorized as having “Severe HRSN,” those who indicated *no* need in any domain as “No HRSN,” and the remaining respondents (i.e., those with one or more needs that did not meet the severe criteria) as “Moderate HRSN.” The resulting scale provides a three-level overall HRSN categorization for each respondent.

### Outcomes

The study outcome was total direct annual healthcare cost. Cost data from 2019 was used because the survey, fielded in 2020, asked about needs experienced in the *previous* year, so it was thought that this would most closely represent need and cost during the same period. Costs were obtained by linking survey data to administrative data by respondents’ medical record numbers. The cost measure reflects all direct healthcare costs and excludes indirect costs, representing accounting estimates of the resources used to deliver the volume and intensity of services provided to patients, including general and specialty care across inpatient, outpatient, and emergency settings. Because of the integrated model of care, the vast majority of utilization occurs within the KP system; however, the costs also include external claims for services provided by non-KP facilities and clinicians (e.g., emergency care at out-of-network hospitals).

### Statistical Analysis

Characteristics of survey respondents were tabulated by HRSN level and overall. Adjusted associations between total healthcare cost and HRSN were assessed via one-way Analysis-of-Variance, with “Moderate HRSN” and “Severe HRSN” each separately compared to the “No HRSN” group.

An indicator variable was used to denote whether the participant completed the survey before or after March 2020, the start of COVID-19-related shutdowns. We tested an interaction term between HRSN and the COVID-19 indicator to determine whether the relationships between HRSN and cost varied between periods. Since that interaction was not significant in any model, we only include the COVID-19 indicator as a covariate.

We constructed multivariable generalized linear models (GLM) to assess the association between HRSN and cost, with the “No HRSN” group as the referent. Control variables included patient demographics (gender, age group, race/ethnicity, KP Region), insurance coverage (insurance type, number of days enrolled in the year), the COVID-19 indicator, level of patient-reported social isolation, and medical risk (DxCG). The DxCG is a risk adjustment score commonly used by insurers to predict healthcare costs of individuals based on past diagnoses and prescription drug coding^27^. The GLMs used gamma distribution with log link function to account for the distribution of the cost outcome. Given the complex relationships between HRSN, insurance coverage, and medical risk, the main results are presented in three models (Table 2) that analyze healthcare cost and HRSN, with varying control variables. Model 1 controlled for limited patient demographics (age group, gender, region), length of insurance enrollment, and the COVID-19 survey indicator. Model 2 added race/ethnicity, insurance type, and social isolation, which were tested in a separate model because HRSNs are commonly known to have strong associations with these factors. Model 3 added an additional control for medical risk using DxCG grouped by quintile, which was separately examined because DxCG is fine-tuned to predict healthcare costs and may also capture elements of social risk. Unless otherwise indicated, all analyses used survey weights accounting for oversampling and non-response using the “Survey” package in R.

## RESULTS

### Study Population and Baseline Characteristics

Overall, 10,226 members (23%) responded between January and September 2020. Characteristics of responders versus non-responders are shown in Appendix 2. After applying survey weights, 52.9% of the population was female. The largest race/ethnicity group was White (41.9%), followed by Hispanic (26.4%), Asian (16.3%), Black/African American (8.3%), Pacific Islander/Native American (4.9%), and Other (2.2%). Most patients (68.3%) were commercially insured through their employer, 7.8% through individual insurance, 16.8% through Medicare, and 5.7% through Medicaid or dual coverage. Of the weighted population, almost half (48.0%) had “No HRSN,” 26.4% had “Moderate HRSN,” and 25.6% had “Severe HRSN” (Table 1). Unweighted distributions are shown separately (Appendix 3).

**Table 1:** Characteristics of Survey Respondents by Level of HRSN After Applying Survey Weights

	**No HRSN** *(48.0% of population)*	**Moderate HRSN** *(26.4% of population)*	**Severe HRSN** *(25.6% of population)*	**Overall** *(100% of population)*
	Weighted % of sample			
Gender				
Female	23.6%	14.9%	14.4%	52.9%
Male	24.4%	11.5%	11.2%	47.1%
Age group				
18–30	7.9%	6.5%	7.2%	21.6%
31–40	7.2%	5.2%	5.0%	17.4%
41–50	7.8%	5.1%	5.3%	18.1%
51–60	9.3%	4.1%	4.2%	17.5%
61–70	8.4%	3.4%	2.2%	13.9%
71 +	7.4%	2.2%	1.8%	11.4%
Race/ethnicity				
White	25.5%	9.6%	6.8%	41.9%
Asian	7.6%	4.5%	4.1%	16.3%
Black/African American	3.1%	1.8%	3.4%	8.3%
Hispanic	9.3%	8.5%	8.6%	26.4%
Pacific Islander/Native American	1.9%	1.4%	1.6%	4.9%
Other	0.6%	0.5%	1.1%	2.2%
Region				
Southern California	16.8%	10.9%	11.2%	38.9%
Northern California	18.5%	9.2%	7.9%	35.6%
Colorado	2.7%	1.3%	0.9%	4.9%
Georgia	1.0%	0.6%	0.8%	2.4%
Hawaii	0.8%	0.6%	0.6%	2.0%
Mid-Atlantic	2.5%	1.2%	2.0%	5.6%
Northwest	3.0%	1.3%	1.1%	5.5%
Washington	2.7%	1.4%	1.1%	5.2%
Type of insurance coverage				
Commercial	32.5%	18.5%	17.2%	68.3%
Individual	3.6%	2.1%	2.2%	7.8%
Medicare	10.5%	3.5%	2.8%	16.8%
Medicaid/dual coverage	0.7%	1.7%	3.2%	5.7%
Other	0.6%	0.6%	0.2%	1.4%
DxCG				
1 st quintile (lowest)	9.9%	5.3%	4.8%	20.0%
2nd quintile	9.3%	5.6%	5.1%	20.1%
3rd quintile	9.7%	5.2%	5.1%	19.9%
4th quintile	9.6%	5.7%	4.8%	20.0%
5th quintile (highest)	9.4%	4.7%	5.9%	20.0%
	Mean (SD) in $			
Total annual healthcare cost*	5501 (16,670)	5419 (18,579)	7030 (50,988)	5871 (29,824)

^*^Calculated using weighted bivariate ANOVA. Wald test to compare cost to that of “No Need” revealed *P* = 0.91 for “Moderate Need” and *P* = 0.13 for “Severe Need”

### Bivariate Association Between HRSN and Total Healthcare Cost

Weighted mean total annual costs were similar in those with “No HRSN” and those with “Moderate HRSN,” with values of $5501 (SD: $16,670) and $5419 (SD: $18,579), respectively. Those with “Severe HRSN” had a mean annual healthcare cost of $7030 (SD: $50,988) (Table 1). These differences were not statistically significant. Associations between need level within each HRSN domain (e.g., Financial Instability, Food Insecurity, Transportation Need, and Housing Instability) and total cost are shown in Appendix 4.

### Multivariable Association Between HRSN and Total Healthcare Cost

**Figure 1:**
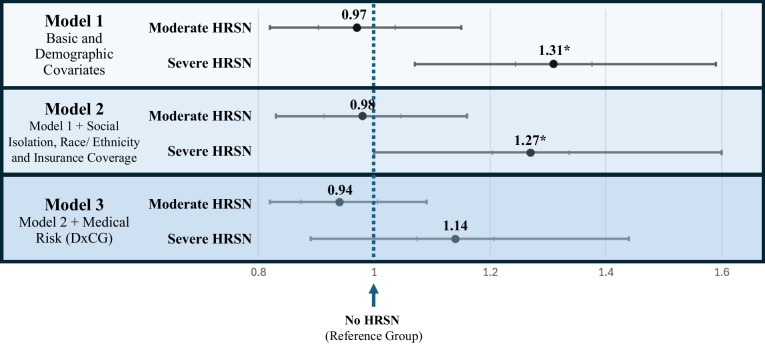
Rate ratio for moderate and severe overall HRSN compared to No HRSN in multivariate models. *Significance for an alpha level of 0.05.

In Model 1, controlling for minimal sociodemographic covariates (age, gender, and region), COVID-19 indicator, and length of enrollment, those with “Severe HRSN” had costs 1.31 (95%CI: 1.07–1.59) times greater than those with “No HRSN.” “Moderate HRSN” showed no significant association with cost (Table 2, Model 1) (Fig. 1).

**Table 2:** Multivariable Regressions: Association of HRSN with Total Annual Healthcare Cost

	**Model 1:** Basic and demographic covariates*	**Model 2:** Model 1 with addition of race/ethnicity, type of insurance coverage, and social isolation*	**Model 3:** Model 2 with addition of medical risk (DxCG)*
**Predictor variables**	Incident rate ratio [IRR] computed from regression output coefficients (95%CI)		
Gender			
Female	1.00 (ref)	1.00 (ref)	1.00 (ref)
Male	0.76 (0.66–0.88)	0.77 (0.67–0.89)	0.99 (0.86–1.13)
Age group			
18–30	1.00 (ref)	1.00 (ref)	1.00 (ref)
31–40	1.15 (0.85–1.54)	1.21 (0.91–1.61)	1.12 (0.85–1.47)
41–50	1.19 (0.9–1.59)	1.27 (0.98–1.66)	0.99 (0.77–1.28)
51–60	1.37 (1.04–1.8)	1.41 (1.09–1.83)	0.80 (0.66–0.96)
61–70	2.17 (1.69–2.79)	2.03 (1.59–2.61)	0.89 (0.74–1.06)
71 +	2.87 (2.21–3.74)	2.43 (1.78–3.31)	0.87 (0.7–1.08)
Race/ethnicity			
White		1.00 (ref)	1.00 (ref)
Asian		0.70 (0.57–0.86)	0.85 (0.70–1.04)
Black/African American		1.23 (1.01–1.48)	1.12 (0.90–1.40)
Hispanic		1.13 (0.91–1.4)	1.04 (0.84–1.29)
Pacific Islander/Native American		1.33 (0.96–1.85)	0.96 (0.77–1.2)
Other		1.07 (0.73–1.58)	0.91 (0.62–1.32)
Social isolation reported^†^			
No need		1.00 (ref)	1.00 (ref)
Moderate need		0.93 (0.77–1.11)	0.99 (0.83–1.18)
Severe need		0.87 (0.71–1.08)	0.97 (0.78–1.22)
Type of insurance coverage			
Commercial		1.00 (ref)	1.00 (ref)
Individual		0.93 (0.73–1.19)	1.00 (0.76–1.32)
Medicare		1.27 (1.04–1.55)	0.95 (0.83–1.09)
Medicaid/dual coverage		1.35 (1.10–1.66)	1.01 (0.83–1.22)
Other		0.74 (0.49–1.12)	0.71 (0.48–1.04)
DxCG			
1 st quintile (lowest)			0.18 (0.13–0.25)
2nd quintile			0.53 (0.44–0.64)
3rd quintile (reference)			1.00 (ref)
4th quintile			2.03 (1.74–2.38)
5th quintile (highest)			5.88 (5.13–6.74)
**Overall HRSN level**			
No HRSN	1.00 (ref)	1.00 (ref)	1.00 (ref)
Moderate HRSN	0.97 (0.82–1.15)	0.98 (0.83–1.16)	0.94 (0.82–1.09)
Severe HRSN	1.31 (1.07–1.59)	1.27 (1.00–1.60)	1.14 (0.89–1.44)

^*^All models included patient region, an indicator for pre- vs. post-COVID-19 survey administration, and number of days in the year insured as covariates, though results are not shown in the table above. The COVID-19 indicator and days insured were not statistically significant in any model

^†^Social isolation was initially one of the core HRSN that the survey evaluated, but was later separated out and recategorized as a covariate rather than considered as a primary HRSN domain given that it was felt that this need, while important, holds less of a relation to material resources than the other four HRSN domains studied

When race/ethnicity, type of insurance, and social isolation were added to the model, the association between “Severe HRSN” and total healthcare cost remained similar, with costs 1.27 (95%CI: 1.00–1.60) times higher than “No HRSN.” “Moderate HRSN” still showed no significant association with cost (Table 2, Model 2) (Fig. 1). With respect to insurance coverage, the relative costs of those with Medicare were 1.27 (95%CI: 1.04–1.55) times the costs of those with commercial insurance, and those for Medicaid/dual coverage were 1.35 (95%CI: 1.10–1.66) times those of the commercially insured population.

In Model 3, medical risk was added to the model as a covariate using DxCG quintiles, with the middle quintile serving as the reference group. DxCG showed a strong positive association with cost, with relative cost increasing with each successive quintile. Those in the 1 st and 2nd quintiles had costs only 18% and 53%, respectively, of those in the 3rd quintile (reference group). Meanwhile, those in the 4th and 5th quintiles had costs that were 203% and 588% those of the reference group. However, in this model, the relationship between "Severe HRSN" and total healthcare cost was not statistically significant (incident rate ratio [IRR] 1.14, 95%CI: 0.89–1.44). “Moderate HRSN” was also not significantly associated with cost (Table 2, Model 3) (Fig. 1).

## DISCUSSION

This cross-sectional study of over ten thousand Kaiser Permanente members examined associations between HRSN and total healthcare cost, as well as how these associations change when considering factors such as insurance coverage and medical risk. The results showed a strong and significant positive relationship between “Severe HRSN” and total healthcare cost. When including medical risk, this association was attenuated. Distinctively, this study explores this association using a broad, patient-reported measure of HRSN in a population that approximates the general US population in terms of demographic characteristics^23^. The data are drawn from an integrated healthcare system, which allows for a more confident measurement of total healthcare cost than may be possible in more fragmented settings. These findings provide a unique addition to the existing body of literature, which largely focuses on specific subpopulations or interventions, and may inform how organizations approach HRSN screening and intervention.

In our analysis, moderate need did not show statistically significant associations with cost, relative to those with no need. However, it is important to note that our cross-sectional study does not follow the progression of patients’ needs over time. Individuals may have moderate needs that ultimately progress in severity, and there may be benefit to early intervention for moderate HRSN. Further, potentially reduced costs represent only one pragmatic motivation for assisting patients with HRSN and do not consider broader health and societal benefits or ethical obligations that healthcare organizations may have to assist patients with HRSN^28^,^33^.

Our study illustrates the challenges of studying the complex relationships between medical risk, social risk, and healthcare cost. Our findings of a large magnitude of association between “Severe HRSN” and cost in models controlling for demographic covariates and insurance status are in line with previous findings establishing associations between HRSN and utilization^5,6^. The attenuation of those results when we introduce a strong control variable for medical risk deserves further investigation, as social risk and medical risk have complex relationships that are often causal and bi-directional in nature. It may be possible that HRSN, even when severe, do not lead to differences in healthcare costs above and beyond the patients’ underlying medical conditions. However, alternative explanations may also be at play. As a risk adjustment scheme, the DxCG essentially uses assigned patient diagnoses and data on prescription drug codes to predict healthcare costs. While some recently developed risk adjustment schemes attempt to incorporate HRSN, the version of DxCG available for this analysis does not directly or separately control for HRSN. Thus, if patients with a given diagnosis also have high levels of HRSN, the DxCG will capture that social risk effect within its index. Consequently, adjusting for DxCG may lead to “over-controlling,” wherein aspects of the independent variable of interest (HRSN) are included in a control variable (DxCG), and true underlying associations between the independent variable and the outcome of interest can be masked. Future studies may be able to provide further clarity on the relationships between medical risk, social risk, and cost. In particular, following patients’ medical and social risks longitudinally may leverage differential changes in each phenomenon over time to better understand independent effects on cost.

Our study may provide insight into challenges of funding efforts to address HRSN interventions. A recent simulation study estimated the costs of screening for and intervening on HRSN in primary care and concluded that costs would likely be higher than what is feasible under current funding mechanisms available to providers^29^. External incentives, collaborations, and financial support may be required if policymakers wish to support further investigation and development of interventions that might have longer-term or non-financial benefits. For example, novel demonstration projects such as Robert Wood Johnson Foundation’s “Bridging the Gap: Reducing Disparities in Diabetes Care” program have shown promising results on how new payment models and deep community partnership can be used to support cross-sectoral, multi-level, and tailored interventions to address patient HRSNs^30,31^. Leveraging these types of partnerships may provide opportunities to address patient HRSNs even in circumstances where the current demonstrable health system return on investment is unclear.

Our findings also highlight the importance of evaluations that are designed up-front to address the potential overlapping measurements of medical and social risks. While our demographic and insurance type models may overestimate potential cost savings by not accounting for differences in disease diagnosis between HRSN groups, our DxCG model—and its potential for over-controlling for social risk—illustrates the need for caution when constructing models where adjustment variables may capture aspects of the independent variable of interest. Given the large potential cost implications of addressing HRSN, it will be essential for health systems to gain greater clarity on this issue through further research.

This study has several additional limitations of note. First, it is unable to make any strong causal inferences, nor does it address whether interventions lead to cost reductions. Instead, our work serves as an indicator of a potential magnitude for cost savings opportunity that may be an important point of information for those considering HRSN interventions. Second, the relationships among the social construct of race, insurance, social need, and healthcare costs are complex. SDoH and HRSN are widely recognized as influencers of racial disparities in health outcomes. Systemic and structural racism are known to lead to higher levels of a variety of social needs^31^^,32^. Healthcare-seeking behaviors of individuals who identify as members of underserved minority groups may be influenced by unfamiliar cultural norms, community concerns, and/or histories of distrust of medical systems, and evaluation and treatment may be influenced by provider bias^33^,^34^. Third, while our study sample is large and diverse, it is nonetheless only comprised of individuals with insurance, and we are unable to comment on the potential relationship between HRSN and cost among the uninsured. Another challenge is that while many low-cost, easily accessible tools are available for HRSN screening, psychometric data on the reliability and validity of these tools is limited^22,34^. Additionally, patient cost sharing (i.e., co-pays and coinsurance) may influence utilization behaviors, though this was outside of the scope of the current study. Lastly, as detailed in the “METHODS,” we studied only direct costs and excluded indirect costs, and so our dollar estimates may not be a full estimate of all resources that go toward the provision of care or the broader impact that HRSNs have on patients’ lives.

While no empirical model can capture the complex and evolving understanding of these constructs perfectly, our analysis aims to provide timely insight into the associations between HRSN and cost, while controlling for the effects of those complicated dynamics as best as our data allow. As we gain a better understanding of these factors, we can begin to envision a system in which HRSN are effectively identified and incorporated into clinical decision making as well as system-wide resource allocation strategies, perhaps viewing HRSN as another aspect of health maintenance or preventive medicine.

## Supplementary Information

Below is the link to the electronic supplementary material.

Supplementary file1 (DOCX 42.8 KB)
