# Supplementary material for: Health-Related Social Needs and Total Healthcare Cost: A Cross-Sectional Study in a Large Integrated Health System
Source: J Gen Intern Med. 2025 Jun 25;41(2):409–16. doi: 10.1007/s11606-025-09647-8 (PMC12894448; doi:10.1007/s11606-025-09647-8)
Supplement: Supplementary file 1 — Supplementary file1 (DOCX 42.8 KB) [file 11606_2025_9647_MOESM1_ESM.docx]

**Appendix 1.** Individual survey question, HRSN level categorization, and source

| **Survey question and HRSN level indicated by response** | **Source of question** |
| --- | --- |
| **Financial instability** | |
| How hard is it for you to pay for the very basics like food, housing, medical care, and heating?   1. Very hard *(Severe Need)* 2. Hard *(Severe Need)* 3. Somewhat hard *(Moderate Need)* 4. Not very hard *(No Need)* 5. Not hard at all *(No Need)* | Center for Medicare and Medicaid The Accountable Health Communities Health-Related Social Needs Screening Tool Supplemental Questions ^36^  Original source: The SWAN sleep study^37^ |
| Thinking about the past 12 months, would you say that at the end of each month you generally ended up with:  More than enough money left over *(No Need)*  Some money left over *(No Need)*  Just enough to make ends meet *(Moderate Need)*  Almost enough to make ends meet *(Severe Need)*  Not enough to make ends meet *(Severe Need)* | Tucker-Seeley Research Laboratory Money-Health Connection Study “Making Ends Meet” items, originally from Conger et al.^38^ |
| **Food insecurity** | |
| Within the past 12 months, you worried whether your food would run out before you got money to buy more.   1. Never true *(No Need)* 2. Sometimes true *(Moderate Need)* 3. Often true *(Severe Need)* | Center for Medicare and Medicaid The Accountable Health Communities Health-Related Social Needs Screening Tool^36,39^  Original source: The Seattle Short Form Validated 2-Item Food Security Screen |
| Within the past 12 months, the food you bought just didn’t last and you didn’t have money to get  more.   1. Never true *(No Need)* 2. Sometimes true *(Moderate Need)* 3. Often true *(Severe Need)* | Center for Medicare and Medicaid The Accountable Health Communities Health-Related Social Needs Screening Tool^36,39^  Original source: The Seattle Short Form Validated 2-Item Food Security Screen^40,41^ |
| Within the past 12 months, it was hard for you to get enough healthy food.   1. Never true *(No Need)* 2. Sometimes true *(Moderate Need)* 3. Often true *(Severe Need)* | Adapted from Your Current Life Situation (YCLS) Question Bank as well as above sources for question format consistency^42^ |
| **Transportation need** | |
| In the past 12 months, has a lack of transportation kept you from medical appointments or from getting medications?   1. Yes *(If “Yes” to this and question below, Severe Need. If “Yes” to only this, Moderate Need)* 2. No *(No Need)* | Adapted from the Center for Medicare and Medicaid The Accountable Health Communities Health-Related Social Needs Screening Tool^36,39^  Original source: PRAPARE Screening Tool^43^ |
| In the past 12 months, has a lack of transportation kept you from meetings, work, or from getting things needed for daily living?   1. Yes *(If “Yes” to this and question above, Severe Need. If “Yes” to only this, Moderate Need)* 2. No *(No Need)* | Adapted from the Center for Medicare and Medicaid The Accountable Health Communities Health-Related Social Needs Screening Tool^36,39^  Original source: PRAPARE Screening Tool^43^ |
| **Housing instability** | |
| In the past 12 months, was there a time when you were not able to pay the mortgage or rent on time?   1. Yes *(Moderate Need)* 2. No *(No Need)* | Children’s Health Watch Housing Instability Vital Sign^44^ |
| In the past 12 months, how many places have you lived?   1. One *(No Need)* 2. Two *(No Need)* 3. Three or more *(Severe Need)* | Children’s Health Watch Housing Instability Vital Sign^44^ |
| In the past 12 months, was there a time when you did not have a steady place to sleep or slept in a shelter (including now)?   1. Yes *(Moderate Need)* 2. No *(No Need)* | Children’s Health Watch Housing Instability Vital Sign^44^ |
| What is your living situation today?   1. You have a steady place to live *(No Need)* 2. You have a place to live today, but you are worried about losing it in the future *(No Need)* 3. You do not have a steady place to live (You are temporarily staying with others, in a hotel, in a shelter, living outside on the street, on a beach, in a car, abandoned building, bus or train station, or in a park) *(Severe Need)* | Adapted from the Center for Medicare and Medicaid The Accountable Health Communities Health-Related Social Needs Screening Tool ^36,39^ |

**Appendix 2.** Characteristics of Responders compared with those of Non-Responders

|  | **Non-Respondents (N=33710)** | **Respondents (N=10226)** | **Overall (N=43936)** |
| --- | --- | --- | --- |
| Gender |  |  |  |
| Female | 15450 (45.8%) | 5813 (56.8%) | 21263 (48.4%) |
| Male | 18260 (54.2%) | 4413 (43.2%) | 22673 (51.6%) |
| Age group |  |  |  |
| 18-30 | 9524 (28.3%) | 1881 (18.4%) | 11405 (26.0%) |
| 31-40 | 5834 (17.3%) | 1591 (15.6%) | 7425 (16.9%) |
| 41-50 | 5473 (16.2%) | 1553 (15.2%) | 7026 (16.0%) |
| 51-60 | 5588 (16.6%) | 1842 (18.0%) | 7430 (16.9%) |
| 61-70 | 4170 (12.4%) | 1923 (18.8%) | 6093 (13.9%) |
| 71+ | 3121 (9.3%) | 1436 (14.0%) | 4557 (10.4%) |
| Region |  |  |  |
| Southern California | 4210 (12.5%) | 1282 (12.5%) | 5492 (12.5%) |
| Northern California | 4131 (12.3%) | 1361 (13.3%) | 5492 (12.5%) |
| Colorado | 4187 (12.4%) | 1305 (12.8%) | 5492 (12.5%) |
| Georgia | 4182 (12.4%) | 1310 (12.8%) | 5492 (12.5%) |
| Hawaii | 4248 (12.6%) | 1244 (12.2%) | 5492 (12.5%) |
| Mid-Atlantic | 4291 (12.7%) | 1201 (11.7%) | 5492 (12.5%) |
| Northwest | 4120 (12.2%) | 1372 (13.4%) | 5492 (12.5%) |
| Washington | 4341 (12.9%) | 1151 (11.3%) | 5492 (12.5%) |
| Type of insurance coverage |  |  |  |
| Commercial | 21748 (64.5%) | 6196 (60.6%) | 27944 (63.6%) |
| Individual | 3455 (10.2%) | 854 (8.4%) | 4309 (9.8%) |
| Medicare | 4320 (12.8%) | 2186 (21.4%) | 6506 (14.8%) |
| Medicaid/Dual coverage | 3312 (9.8%) | 763 (7.5%) | 4075 (9.3%) |
| Other | 875 (2.6%) | 227 (2.2%) | 1102 (2.5%) |

**Appendix 3.** Characteristics of Surveyed Members by Level of HRSN, Unweighted *(see Table 1 for weighted)*

|  | **No HRSN** **(N=4888)** | **Moderate HRSN** **(N=2687)** | **Severe HRSN** **(N=2651)** | **Overall (N=10226)** |
| --- | --- | --- | --- | --- |
| Gender |  |  |  |  |
| Female | 2720 (55.6%) | 1539 (57.3%) | 1554 (58.6%) | 5813 (56.8%) |
| Male | 2168 (44.4%) | 1148 (42.7%) | 1097 (41.4%) | 4413 (43.2%) |
| Age group |  |  |  |  |
| 18-30 | 651 (13.3%) | 596 (22.2%) | 634 (23.9%) | 1881 (18.4%) |
| 31-40 | 641 (13.1%) | 475 (17.7%) | 475 (17.9%) | 1591 (15.6%) |
| 41-50 | 625 (12.8%) | 427 (15.9%) | 501 (18.9%) | 1553 (15.2%) |
| 51-60 | 890 (18.2%) | 457 (17.0%) | 495 (18.7%) | 1842 (18.0%) |
| 61-70 | 1142 (23.4%) | 438 (16.3%) | 343 (12.9%) | 1923 (18.8%) |
| 71+ | 939 (19.2%) | 294 (10.9%) | 203 (7.7%) | 1436 (14.0%) |
| Race/ethnicity |  |  |  |  |
| White | 3077 (63.0%) | 1194 (44.4%) | 940 (35.5%) | 5211 (51.0%) |
| Asian | 542 (11.1%) | 389 (14.5%) | 339 (12.8%) | 1270 (12.4%) |
| Black/African American | 500 (10.2%) | 340 (12.7%) | 526 (19.8%) | 1366 (13.4%) |
| Hispanic | 504 (10.3%) | 512 (19.1%) | 520 (19.6%) | 1536 (15.0%) |
| Pacific Islander / Native American | 176 (3.6%) | 180 (6.7%) | 245 (9.2%) | 601 (5.9%) |
| Other | 89 (1.8%) | 72 (2.7%) | 81 (3.1%) | 242 (2.4%) |
| Region |  |  |  |  |
| Southern California | 551 (11.3%) | 373 (13.9%) | 358 (13.5%) | 1282 (12.5%) |
| Northern California | 637 (13.0%) | 390 (14.5%) | 334 (12.6%) | 1361 (13.3%) |
| Colorado | 727 (14.9%) | 330 (12.3%) | 248 (9.4%) | 1305 (12.8%) |
| Georgia | 580 (11.9%) | 320 (11.9%) | 410 (15.5%) | 1310 (12.8%) |
| Hawaii | 508 (10.4%) | 370 (13.8%) | 366 (13.8%) | 1244 (12.2%) |
| Mid-Atlantic | 527 (10.8%) | 272 (10.1%) | 402 (15.2%) | 1201 (11.7%) |
| Northwest | 726 (14.9%) | 342 (12.7%) | 304 (11.5%) | 1372 (13.4%) |
| Washington | 632 (12.9%) | 290 (10.8%) | 229 (8.6%) | 1151 (11.3%) |
| Type of insurance coverage |  |  |  |  |
| Commercial | 2930 (59.9%) | 1659 (61.7%) | 1607 (60.6%) | 6196 (60.6%) |
| Individual | 369 (7.5%) | 249 (9.3%) | 236 (8.9%) | 854 (8.4%) |
| Medicare | 1366 (27.9%) | 453 (16.9%) | 367 (13.8%) | 2186 (21.4%) |
| Medicaid/Dual coverage | 104 (2.1%) | 257 (9.6%) | 402 (15.2%) | 763 (7.5%) |
| Other | 119 (2.4%) | 69 (2.6%) | 39 (1.5%) | 227 (2.2%) |
| DxCG |  |  |  |  |
| 1^st^ quintile (lowest) | 732 (15.0%) | 474 (17.6%) | 430 (16.2%) | 1636 (16.0%) |
| 2^nd^ quintile | 819 (16.8%) | 490 (18.2%) | 414 (15.6%) | 1723 (16.8%) |
| 3^rd^ quintile | 1007 (20.6%) | 493 (18.3%) | 513 (19.4%) | 2013 (19.7%) |
| 4^th^ quintile | 1165 (23.8%) | 612 (22.8%) | 585 (22.1%) | 2362 (23.1%) |
| 5^th^ quintile (highest) | 1158 (23.7%) | 615 (22.9%) | 708 (26.7%) | 2481 (24.3%) |

**Appendix 4.** Mean total annual healthcare cost by need level within each HRSN domain, Total cost in $ (Standard Deviation) *(Weighted percent of population)*

|  | **No need in domain** | **Moderate need in domain** | **P-value*** | **Severe need in domain** | **P-value*** |
| --- | --- | --- | --- | --- | --- |
| Financial instability | 5,306 (16,048) *(55%)* | 6,098 (19,543)  *(28%)* | 0.30 | 7,391 (62,552)  *(16%)* | 0.12 |
| Food insecurity | 5,432 (16,632)  *(68%)* | 7,542 (53,415)  *(24%)* | 0.06 | 4,471 (11,783)  *(7%)* | 0.09 |
| Transportation need | 5,677 (30,063)  *(93%)* | 8,810 (29,740)  *(4%)* | 0.18 | 8,391 (19,918)  *(3%)* | 0.07 |
| Housing instability | 5,514 (17,365)  *(83%)* | 4,947 (12,322)  *(3%)* | 0.57 | 8,287 (68,468)  *(14%)* | 0.08 |

*Wald test used to calculate p-value from simple weighted gamma with log link GLM for difference in total cost between 3 weighted groups without winsorization.

**References for Appendix 1**:

36. The Accountable Health Communities Health-Related Social Needs Screening Tool. [https://www.cms.gov/priorities/innovation/files/worksheets/ahcm-screeningtool.pdf](https://urldefense.com/v3/__https:/www.cms.gov/priorities/innovation/files/worksheets/ahcm-screeningtool.pdf__;!!F9wkZZsI-LA!BXWPIm8w-jXfKujTeDTML2ZfO8O3YviQhDHnOd9lx2N8BfuVOSkX93CNucnpg57OvPyLupd4RjgRY_v43zfg7i5s7boqm92HUA$)

37. Mh H, Ka M, Hm K, et al. Race and financial strain are independent correlates of sleep in midlife women: the SWAN sleep study. Sleep. 2009;32(1). Accessed April 9, 2024.

[https://pubmed.ncbi.nlm.nih.gov/19189781/](https://urldefense.com/v3/__https:/pubmed.ncbi.nlm.nih.gov/19189781/__;!!F9wkZZsI-LA!BXWPIm8w-jXfKujTeDTML2ZfO8O3YviQhDHnOd9lx2N8BfuVOSkX93CNucnpg57OvPyLupd4RjgRY_v43zfg7i5s7bqbob5LxQ$)

38. Conger RD, Conger KJ, Matthews LS, Elder GH. Pathways of Economic Influence on Adolescent Adjustment. Am J Community Psychol. 1999;27(4):519-541. [https://doi.org/10.1023/A:1022133228206](https://urldefense.com/v3/__https:/doi.org/10.1023/A:1022133228206__;!!F9wkZZsI-LA!BXWPIm8w-jXfKujTeDTML2ZfO8O3YviQhDHnOd9lx2N8BfuVOSkX93CNucnpg57OvPyLupd4RjgRY_v43zfg7i5s7bpavgwmzA$)

39. Billioux A, Verlander K, Anthony S, Alley D. Standardized screening for health-related social needs in clinical settings: The accountable health communities screening tool. Natl Acad Med.
Published online May 30, 2017. [https://nam.edu/wp-content/uploads/2017/05/Standardized-Screeningfor-Health-Related-Social-Needs-in-Clinical-Settings.pdf](https://urldefense.com/v3/__https:/nam.edu/wp-content/uploads/2017/05/Standardized-Screeningfor-Health-Related-Social-Needs-in-Clinical-Settings.pdf__;!!F9wkZZsI-LA!BXWPIm8w-jXfKujTeDTML2ZfO8O3YviQhDHnOd9lx2N8BfuVOSkX93CNucnpg57OvPyLupd4RjgRY_v43zfg7i5s7bpy3fdVZQ$)

40. Radandt NE, Corbridge T, Johnson DB, Kim AS, Scott JM, Coldwell SE. Validation of a Two-Item Food Security Screening Tool in a Dental Setting. J Dent Child Chic Ill. 2018;85(3):114-119.

41. Hager ER, Quigg AM, Black MM, et al. Development and validity of a 2-item screen to identify families at risk for food insecurity. Pediatrics. 2010;126(1):e26-32. [https://doi.org/10.1542/peds.2009-](https://urldefense.com/v3/__https:/doi.org/10.1542/peds.2009-__;!!F9wkZZsI-LA!BXWPIm8w-jXfKujTeDTML2ZfO8O3YviQhDHnOd9lx2N8BfuVOSkX93CNucnpg57OvPyLupd4RjgRY_v43zfg7i5s7bqGlCcDww$)
3146

42. LaForge K, Gold R, Cottrell E, et al. How 6 Organizations Developed Tools and Processes for Social Determinants of Health Screening in Primary Care: An Overview. J Ambulatory Care Manage.
2018;41(1):2. [https://doi.org/10.1097/JAC.0000000000000221](https://urldefense.com/v3/__https:/doi.org/10.1097/JAC.0000000000000221__;!!F9wkZZsI-LA!BXWPIm8w-jXfKujTeDTML2ZfO8O3YviQhDHnOd9lx2N8BfuVOSkX93CNucnpg57OvPyLupd4RjgRY_v43zfg7i5s7bqOdX8WFg$)

43. The PRAPARE Screening Tool | PRAPARE. Accessed April 9, 2024. [https://prapare.org/the-praparescreening-tool/](https://urldefense.com/v3/__https:/prapare.org/the-praparescreening-tool/__;!!F9wkZZsI-LA!BXWPIm8w-jXfKujTeDTML2ZfO8O3YviQhDHnOd9lx2N8BfuVOSkX93CNucnpg57OvPyLupd4RjgRY_v43zfg7i5s7boOpxWq9g$)

44. Children’s Health Watch. Final: 2018: children’s health watch survey [Survey]. Published online 2018.
